# “Mess-o-stomosis”: a matter of malpractice rather than awkwardness

**DOI:** 10.1186/1471-2482-13-S1-A37

**Published:** 2013-09-16

**Authors:** Gianluca Pellino, Guido Sciaudone, Giuseppe Candilio, Antonio Camerlingo, Rosa Marcellinaro, Federica Rocco, Serena De Fatico, Silvestro Canonico, Francesco Selvaggi

**Affiliations:** 1 Unit of General and Geriatric Surgery, Second University of Naples, Italy

## Introduction

Postoperative staple line leaks and bleeding are the most common reasons for complications in surgical procedures that involve organ resection [1]. Trans-anal stapling devices are nowadays widely used; however, complications may still affect a considerable rate of patients, especially when staplers are activated by inexperienced surgeons [2]. This potentially leads to catastrophic consequences in frail patients. We herein describe the case of a patient referred to our Unit suffering from a complication due to an inappropriate use of a trans-anal stapling device.

## Case report

A 67-year-old woman came to our observation with an history of left ovariectomy and hysterectomy performed for a tubo-ovarian abscess with pelvic sepsis in an Obstetrics and Gynaecology Unit. After three weeks, she needed right ovariectomy because of a relapse of the abscess. A lesion to the sigmoid colon occurred during debridement of adhesions. For this reason, surgeons performed a sigmoidal resection and trans-anal colorectal anastomosis with a circular stapler; a colostomy was fashioned in left lower quadrant. The stoma was closed six months later. Few days after colostomy closure, the patient suffered from faecal discharge from the vagina and was referred to our Unit.

**Figure 1:**
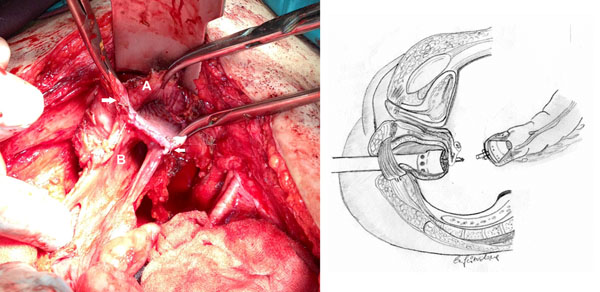
**Left:** Intraoperative picture, showing the opened vagina (A) and rectum (B). The Mayo anterior retractor is pulling up the bladder. Agraphes are clearly identified, both in vagina and rectum (white arrows). **Right:** The drawing schematizes the wrong procedure performed at the time of the first colorectal anastomosis. The staple-gun is passed through the posterior wall of the vagina and rectal pursue string, then it is connected to the colonic stump: firing causes the formation of a vaginal-colonic cloaca.

On exam we found her to have a large defect in the posterior vaginal wall communicating with the rectal lumen, thus determining a common “*cloaca*” involving both structures. At laparotomy we found the vagina to be anastomosed to the colon and rectum, and several lines of *agraphes* were clearly be identified in both (figure 1). Also, the cervix had not been removed. We performed resection of the cervix, closure of the vagina, resection of the damaged colon and stapled colorectal anastomosis, carefully checking not to make the same mistake. Integrity of the anastomosis was checked with intraoperative endoscopy. She was discharged on post-operative day ten without significant complications.

## Discussion

Stapling devices to perform digestive anastomoses have gained wide popularity in the last years. They are reported to be time-saving and to make easier procedures otherwise very difficult to perform (i.e. low pelvic anastomoses)[2]. This has to be counterbalanced with the need for extensive experience to achieve advantages from the technique and with potential complications of using a stapling device. These include perforation, incomplete cutting of the intestinal ends, postoperative haemorrhage, rectal diverticula[3], leakage and stenosis of the anastomotic site[2-5]. Antonsen et al. [4] reported a rate of recto-vaginal fistula as high as 2.2%. In very low anastomoses this could be due to an inadequate retraction of the vagina, but the posterior vaginal wall can slip under the retractor, being caught by the device during firing [5]. Contrastingly, trans-anal stapled anastomoses are often performed by trainees.

In our patient, the complication probably resulted from a combination of several elements: first, surgeons may have not well identified, prepared and retracted the vagina when performing the anastomosis; second, the tissue doughnuts were not inspected after firing. We recommend to follow such easy-to-perform steps; moreover, it could be useful to have a perineal assistant introducing a finger through the vagina while firing in complex cases.

## Conclusions

We feel like technology can make almost everyone able to perform complex or advanced procedures more easily; however, if it is not knowledge-driven surgical disaster are very likely to happen. Technical advancements cannot alter a faulty procedure and are not to be intended for a “*todos caballeros*” policy. Rather, these should be advocated only after careful evaluation of their potential complications, and carried out if one is able to face them properly.

## Competing interests

Authors have no competing interests to disclose.

## Authors’ contributions

GP designed the study, and wrote the draft of the manuscript. GS and GC collected data and participated in the drafting. GS made the drawing. SDF, AC, RM and FR collected and analyzed data. SC and FS conceived of the study, participated in its design and coordination and helped to draft the manuscript. FS performed surgical procedure in our Unit as operating surgeon. All authors read and approved the final manuscript.
